# Evaluation of the use of individualized patient care plans in frequent emergency department visitors with pain complaints

**DOI:** 10.1186/s12245-022-00440-6

**Published:** 2022-08-22

**Authors:** Fred Blind, James Melton, Juliana Karp, Karen Oldano, Karen Homa, Alexandra Blanco, Reanna Leoni, Anthony Pazanese

**Affiliations:** 1 Lakeland Regional Medical Center, Lakeland, FL USA; 2 Nova Southeastern University Dr. Kiran C. Patel College of Osteopathic Medicine, Fort Lauderdale, USA

**Keywords:** Pain, Medical management, Opiod, Care plans, Frequent emergency department visitors

## Abstract

**Background:**

Pain is one of the most common complaints that patients present to the emergency department for; emergency medicine providers are tasked with providing appropriate pain relief while simultaneously limiting the risk of personal and societal harm that may result from opioid misuse. The Lakeland Regional Medical Center developed a medical management program that identified frequent emergency department visitors with a chief complaint of pain. Individualized care plans were developed for these patients. A retrospective review was then conducted to assess the efficacy of these care plans in reducing the number of emergency department visits for pain-related complaints by the patients entered into the medical management program.

**Results:**

There were 294 patients; 65% were male, and the median age was 41 (interquartile range: 33 to 51). A total of 80% percent of the patients were white, and the payors were as follows: 53% were self-pay, 42% were government programs, and 5% had private insurance. The three most common chronic pain complaints were 39% abdominal pain, 24% back/neck pain, and 23% headache/migraine (patients could have more than one area of pain). A total of 60% of the patients had a primary care provider, and another 18% had a pain management provider in addition to primary care.

Post plan admissions were significantly reduced to a median of 1 (*IQR* 0 to 3) with the Wilcoxon signed-rank test’s *p*-value of less than 0.001.

**Conclusion:**

The authors describe their experience with a quality improvement initiative that identifies frequent emergency department visitors with a chief complaint of pain and provides individualized care plans to these patients. The goals of the program are to improve patient’s quality and consistency of care, through interventions that eliminate the prescribing of opioids while providing non-opioid alternatives.

## Introduction

Emergency departments (ED) provide care for both nonurgent and life-threatening conditions and disease states. In 2018, the Centers for Disease Control and Prevention reported 130 million emergency room visits throughout the USA [1]. This statistic encompasses total visits and does not distinguish among patients who had a single visit from those who had multiple visits over the course of the year. A 2010 literature search found frequent ED use definitions ranged from 2 to 12 visits per year [2]. The most common definition found in the literature of a frequent ED visitor is one who visits the ED at least 4 times per year. Utilizing this definition, frequent ED visitors account for 4.5–8% of all ED patients and are estimated to represent approximately 21–28% of all ED visits [2]. These patients generally report poorer physical health compared to patients using the ED less often.

Among all patients presenting to the ED, pain is typically a major complaint or symptom with report of up to 42% of all ED visits related to painful conditions [3]. The American College of Emergency Physicians therefore issued a policy statement in 2012 on the prescribing of opioids for adult patients in the ED [3]. Within this statement, it is noted that for patients aged 10–19 and 20–29 years old, emergency providers have been found to rank third among all specialties in terms of number of opioid prescriptions written and ranked fourth for 30–39 years old. This translates to approximately 12% of total opioid prescriptions written. Since the publication of this position statement, availability and utilization of individual state prescription drug monitoring programs for reporting controlled substance dispensing have increased, allowing providers to make more informed prescribing decisions. The policy statement stresses the importance and obligation of the emergency medicine provider in dispensing appropriate pain relief while simultaneously limiting the risk of personal and societal harm that may result from opioid misuse.

The Lakeland Regional Medical Center holds the distinction of being the busiest single site ED in the USA with 210,020 reported visits in 2017 [4, 5]. Beginning in 2008, the medical management initiative was implemented to identify frequent ED visitors with a chief complaint of pain and provide individualized care plans to those patients. The program seeks to improve patient’s quality and consistency of care, through interventions that eliminate the prescribing of opioids and provide non-opioid alternatives.

The purpose of this study was to retrospectively evaluate whether the multidisciplinary team initiative produced a 25% reduction in those patients identified as frequent visitors to the Lakeland Regional Medical Center Emergency Department.

## Methods

### Intervention

Patients in our medical management initiative were suggested by physicians, nurses, and pharmacists working in the ED and referred to the committee. These patients were reviewed by a multidisciplinary team made up of physicians (hospital medicine and emergency medicine), nurses, pharmacists (ED pharmacist and pain pharmacist), social work and behavioral health, and legal representation. Every patient that was referred was reviewed by the multidisciplinary committee. Most patients referred were entered into the program after all patient details were reviewed. There was also tracking of number of visits, number of CT scans ordered for the same patient complaint, and number of opioid prescriptions written. The committee met quarterly with additional meetings as needed to review new patients.

Utilizing chart review for the most frequent complaints, a plan was developed to address the patient’s care. These plans utilized non-opioid alternatives to address the patient’s pain. If patients were dissatisfied with their medical management plan, they were told to return at 9 AM on the next business day to discuss the plan with the ED pharmacist. Once a plan was finalized for a patient, an easily identifiable icon was attached to their electronic medical record. The medical provider would open the icon and could easily identify the detailed plan for the patient’s care. Every effort was made to encourage advanced practice providers and physicians to follow the treatment plan in order to provide continuity in the care of the patient. Patients were allowed and encouraged to discuss their plan; however, no changes were allowed or negotiated during an ED visit. The committee reviewed plans on an ongoing basis to keep them updated, and any changes were updated in the electronic medical record.

### Measures

All patients included in the intervention were listed in a spreadsheet, and clinicians reviewed the EMR for the following data elements: (1) demographic characteristics: age, gender, race/ethnicity, residence type, and insurance; (2) clinical: patient’s chief complaint, past medical history, substance abuse history, primary care provider, and pain management provider; and (3) number of ED visits: pre plan and post plan and number of admissions: pre plan and post plan. The time frame was the same for both pre and post, which was 1 year.

Measures were summarized using categories, such as age were grouped as follows: 0 to 17, 18 to 24, 25 to 34, 35 to 44, 45 to 54, and 55 to 64. Patients could have more than one chief complaint; thus, complaints were coded into groups of headache/migraine, chest pain, abdominal pain, back/neck pain, musculoskeletal/extremity pain, and other pain. Patients could have more than one condition or disease for their past medical history, and the following was extracted: depression, anxiety, bipolar, pain, hypertension, diabetes, chronic obstructive pulmonary disease, coronary artery disease, headache/migraine, back pain, and abdominal pain. Substance history was coded for patients that reported tobacco, alcohol, opioids, and illicit drugs. The change in number of visits/admissions was calculated by post plan minus pre plan (negative number was a decrease in post visits/admission compared to pre).

### Analysis

The measures were summarized to describe the patients, and two types of statistical tests were used to determine whether post visits were significantly reduced compared to pre. To determine the difference between pre and post plan for the number of visits/admissions, the Wilcoxon signed-rank test was used. Additionally, number of visits/admissions was categorized into groups, and the McNemar test was used to determine differences between pre and post plan proportions. A *p*-value of less than 0.05 was considered significant.

## Results

The medical management initiative began at the Lakeland Regional Medical Center in 2008. Data for this study was collected from 2013 through March 2020. The process of entry into the initiative remains very much the same.

### Patient demographic characteristics and clinical variables

Table 1 summarizes the patient demographic characteristics and clinical variables. For the 294 patients, 65% were male, and the median age was 41 (interquartile range 33 to 51). A total of 80% of the patients were white, and the payors were as follows: 53% were self-pay, 42% were government programs, and 5% had private insurance. A total of 92% had a home as their place of residence. The three most common chronic pain complaints were 39% abdominal pain, 24% back/neck pain, and 23% headache/migraine (patients could have more than one area of pain). For past medical history, most patients had a condition or disease in which 78% of the patients had 3 or more. A total of 37% of the patients had hypertension, 20% had diabetes, 16% had chronic obstructive pulmonary disease, 18% had headache/migraine, 27% had abdominal pain, and less than 1/4th of the patients had depression, anxiety, and/or bipolar. Two-thirds of the patients had a substance abuse history in which almost half (47%) were tobacco users and 30% reported alcohol. A total of 60% of the patients had a primary care provider, and another 18% had a primary and also had a pain management provider.

**Table 1:** Patient demographic characteristics and clinical variables

	*n* (%)
Patient demographic characteristics	
Gender	
Male	191 (65%)
Female	103 (35%)
Age (years)	
0 to 17	2 (0.7%)
18 to 24	17 (6%)
25 to 34	65 (22%)
35 to 44	75 (26%)
45 to 54	79 (27%)
55 to 64	31 (11%)
65 and older	25 (9%)
Race and ethnicity	
White	234 (80%)
Black	47 (16%)
Hispanic	13 (4%)
Payor	
Self-pay	156 (53%)
Government	124 (42%)
Private	14 (5%)
Residence	
Home	271 (92%)
Homeless	17 (6.0%)
ALF/SNF/LTAC	6 (2.0%)
Clinical variables	
Chief complaint	
Pain location^a^	
Abdominal	116 (39%)
Back/neck	72 (24%)
Headache/migraine	69 (23%)
Chest	39 (13%)
MSK/extremity	28 (10%)
Other	21 (7%)
No. of conditions/diseases	
None	5 (1.7%)
1 or 2	61 (21%)
3 or 4	90 (31%)
5 to 7	92 (31%)
8 or more	46 (16%)
Condition/disease^a^	
Hypertension	110 (37%)
Abdominal pain	79 (27%)
Anxiety	65 (22%)
Diabetes	60 (20%)
Depression	56 (19%)
Bipolar	54 (18%)
Headache/migraine	53 (18%)
COPD	48 (16%)
Back pain	48 (16%)
CAD	19 (6%)
Social history	
Substance abuse history	
Yes	195 (66%)
No	99 (34%)
Drug^a^	
Tobacco	137 (47%)
Illicit drugs	98 (33%)
Alcohol	88 (30%)
Opioids	21 (7%)
Provider type	
PCP	176 (60%)
Pain	8 (3%)
PCP and pain	53 (18%)
No PCP and/or pain	57 (19%)

^a^Patients could have more than one. *COPD* is chronic obstructive pulmonary disease; *ALF* is assisted living facility; *SNF* is skilled nursing facility; *LTAC* is long-term acute care; *MSK* is musculoskeletal; *PCP* is primary care provider

**Fig. 1:**
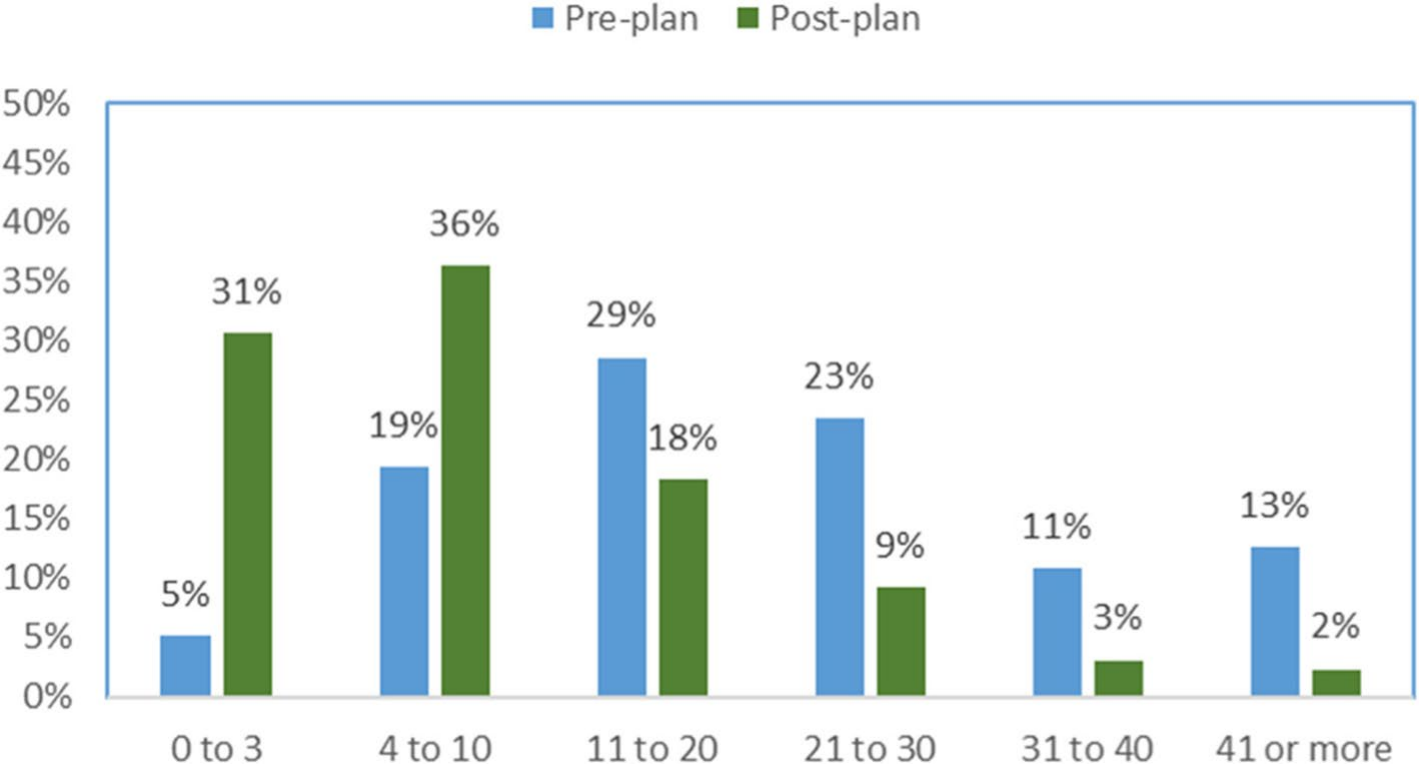
Percentage of patients’ emergency department visits pre and post plan

Table 2 lists the results for pre and post plan ED visits and admissions. For the 294 patients, the median number of preplan ED visits was 20 (interquartile [IQR] range 11 to 29). Post plan visits were significantly reduced to a median of 6 (*IQR* 3 to 13) with the Wilcoxon signed-rank test’s *p*-value of less than 0.001. There were 6779 preplan ED visits, which had a 56% reduction of post plan visits to 3015. A total of 86% of the patients decreased their ED visits by 1 or more, and 67% of the patients decreased their visits by 6 or more. Figure 1 shows the percentage of patients’ pre and post plan ED visits relative to 6 groups (0 to 3, 4 to 10, 11 to 20, 21 to 30, 31 to 40, and 41 or more). The figure shows a higher proportion of patients had 11 or more visits, which shifted in post plan to a higher proportion of patients having 10 or less visits. The McNemar tests to determine differences in proportions were significantly different (*p* < 0.0001, e.g., 2 × 2 table of 0 to 3 visits versus 4 or more visits, 2 × 2 table of 0 to 10 versus 11 or more).

**Table 2:** Pre and post plan summary results of emergency visits and hospital admissions

	Emergency department			
	Pre	Post	Post minus pre	*p*-value*
Median (IQR)	20 (11 to 29)	6 (3 to 13)	−11 (−18 to −3)	< 0.001
Groups *n* (%)				
0 to 3	15 (5%)	90 (31%)		< 0.0001
4 to 10	57 (19%)	107 (36%)		< 0.0001
11 to 20	84 (29%)	54 (18%)		< 0.0001
21 to 30	69 (23%)	27 (9%)		< 0.0001
31 to 40	32 (11%)	9 (3.1%)		< 0.0001
41 or more	37 (13%)	7 (2.4%)		
Change group *n* (%)				
Increase of 1 or more			31 (11%)	
No change			10 (3.4%)	
Decrease of 1 to 5			57 (19%)	
Decrease 6 to 15			103 (35%)	
Decrease of 16 to 25			53 (18%)	
Decrease of 26 or more			40 (14%)	
	Hospital admissions			
	Pre	Post	Post minus pre	*p*-value*
	3 (1 to 7)	1 (0 to 3)	−1 (−4 to 0)	< 0.001
Groups *n* (%)				
Zero	69 (23%)	142 (48%)		< 0.0001
1 to 2	72 (24%)	72 (24%)		< 0.0001
3 to 5	59 (20%)	42 (14%)		< 0.0001
6 to 10	58 (20%)	24 (8%)		0.0001
11 or more	36 (12%)	14 (5%)		
Change group *n* (%)				
Increase of 1 or more			41 (14%)	
No change			79 (27%)	
Decrease of 1 or 2			66 (22%)	
Decrease of 3 to 5			53 (18%)	
Decrease of 6 to 10			38 (13%)	
Decrease of 11 or more			17 (5.8%)	

*IQR* is interquartile range. *p-value to determine differences between pre and post was the Wilcoxon signed-rank test and to determine differences in proportions was the McNemar test

**Fig. 2:**
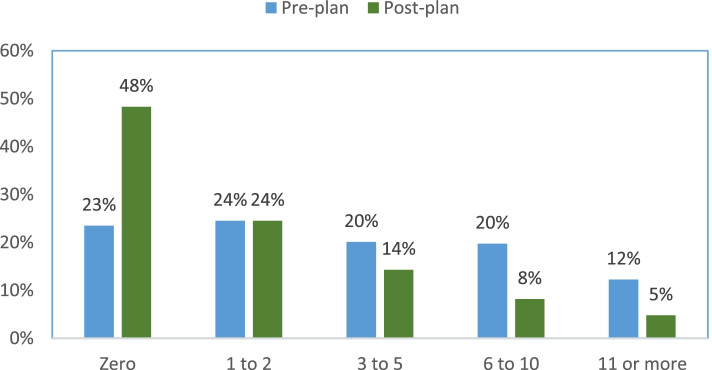
Percentage of patients’ hospital admissions pre and post plan

Listed in Table 2, the median number of preplan admissions was 3 (interquartile range 1 to 7). Post plan admissions were significantly reduced to a median of 1 (*IQR* 0 to 3) with the Wilcoxon signed-rank test’s *p*-value of less than 0.001. There were 1401 admissions pre plan, which had a 53% reduction to 655 post plan admissions. A total of 59% of the patients decreased their admissions to 1 or more, and 38% of the patients decreased admissions to 3 or more. Figure 2 shows the percentage of pre and post plan patients’ admissions relative to 5 groups (0, 1 to 2, 3 to 5, 6 to 10, and 11 or more). The figure shows a higher proportion of patients had 3 or more admissions which shifted in post plan to a higher proportion of no admissions. The McNemar tests to determine differences in proportions were significantly different (*p* < 0.0001, e.g., 2 × 2 table of 0 admissions versus 1 or more admissions, 2 × 2 table of 0 to 2 versus 3 or more).

## Discussion

At the Lakeland Regional Medical Center, we developed and implemented individualized care plans for frequent ED users with pain-related chief complaints. Our goal was achieved as we had a 56% reduction in ED visits and 53% reduction in admissions for 294 patients entered into the medical management initiative.

Multiple previous studies have shown that individualized care plans can reduce ED visits in frequent users [6–8]. To our knowledge, this is the longest data collection of patients referred into a medical management program.

Our study supports the results previously reported. In our study, abdominal pain was overwhelmingly the most frequent pain-related chief complaint, followed by back/neck pain and headache. Similar to other studies [9], self-pay and government insurance patients were encountered most frequently (95%). Also similar to other studies, there was a prevalence of substance abuse history, psychiatric history, and the presence of multiple comorbidities.

Unlike other studies, our frequent user population was predominantly male and white [6, 10]. Also unlike other studies [10], homelessness did not play a significant role. This may be accounted for by the fact that the Lakeland Regional Medical Center serves a semirural patient population encompassing a large geographic area. Even in this unique environment, 60% of patients had access to a primary care physician. In addition, while other studies focused on multiple diagnoses, our study focused only on patients with pain complaints. However, our study supports the results previously reported in a smaller patient population with pain complaints [11].

Previous studies have employed various robust strategies to reduce emergency department visits in frequent visitors. These have included involvement of primary care physicians and social workers. Coordination of care and arrangement of outpatient visits and referral to specialist have been successful. Our individual care plans were not as highly integrated with outpatient resources as some previous studies. The use of a relatively simple individualized care plan focused on frequent users with pain complaints was quite successful in reducing visits. Our 56% reduction in emergency department visits was higher than previously reported. This may be because of our focus on pain complaints only.

A limitation of this study is that although we reduced visits at the Lakeland Regional Medical Center, we do not know if patients went elsewhere for their care or reduced visits secondary to an improved pain management regimen. Further research could focus on this unknown as well as studying how many opiate prescriptions were written pre and post plan. Another limitation is the potential for bias in patient selection. The use of a large multidisciplinary committee with representation from many departments allowed for numerous perspectives to be evaluated on each patient.

## Conclusion

In this longitudinal study in the single busiest emergency department in the USA, the authors describe a successful quality improvement intervention that reduced the overall number of hospital admissions for pain-related complaints. Thoughtful identification of patients at risk coupled with individualized care plans may help to combat opioid misuse.

## Data Availability

All data generated or analyzed during this study are included in this published article.

## Abbreviations

ED: Emergency department
COPD: Chronic obstructive pulmonary disease
ALF: Assisted living facility
SNF: Skilled nursing facility
LTAC: Long-term acute care
MSK: Musculoskeletal
PCP: Primary care provider
